# Flipping plankton

**DOI:** 10.1073/pnas.2603416123

**Published:** 2026-05-27

**Authors:** Bridget S. Wade, Paul N. Pearson, David J. King, Valeria Luciani

**Affiliations:** ^a^Department of Earth Sciences, University College London, London WC1E 6BT, United Kingdom; ^b^Dipartimento di Fisica e Scienze della Terra, Università di Ferrara, Ferrara 44122, Italy

**Keywords:** coiling, evolution, population sweeps, speciation

## Abstract

Sudden, global reversals in shell coiling direction are a striking and recurrent feature in the fossil record of planktonic foraminifera (marine zooplankton), yet their evolutionary significance has been a mystery. Because coiling direction is a simple, binary character, such shifts have often been interpreted as environmentally induced phenotypic responses rather than indicators of evolutionary change, although it is increasingly evident that genetic variants can have different coiling preferences. Here, we synthesize recent evidence from multiple case studies spanning the Eocene to the Recent (the last 56 Mya). Coiling flips occur on timescales of thousands of years or less, across diverse taxa and ocean basins, far too abruptly to be explained by gradual trait evolution. Instead, these rapid, synchronous coiling reversals may signal cryptic speciation and episodic population sweeps, associated with distinct habitat preferences and water mass distributions. In most cases such replacements would leave little trace in the fossil record, but when competing groups differ in coiling preference, a dramatic and geologically abrupt coiling reversal becomes visible. These findings challenge the assumption that reproductive isolation alone delimits species in planktonic foraminifera and instead supports a model of ecological speciation mediated by habitat partitioning in the open ocean. Shell coiling direction thus serves not as an adaptive trait, but as a fortuitous marker of hidden evolutionary dynamics shaping marine microplankton diversity.

Chirality (directional asymmetry) is widespread in nature, from snail shells to flower petals. Despite its simplicity as an easy to document, binary trait, it often hides deeper biological complexities. The chirality of one group of organisms has recently been attracting attention regarding what it can tell us about evolutionary mechanisms. These are the planktonic foraminifera (“forams”), single-celled zooplankton that secrete minute, calcitic spiral shells (Fig. 1). Though tiny, they accumulate in vast numbers on the sea floor, forming major components of ocean sediments. Typical population sizes are vast, with a standing stock of ~10^16^ individuals of a species. The sediments accumulate, preserving a remarkably complete fossil record, capturing information about oceanographic change and plankton evolution.

**Fig. 1.**
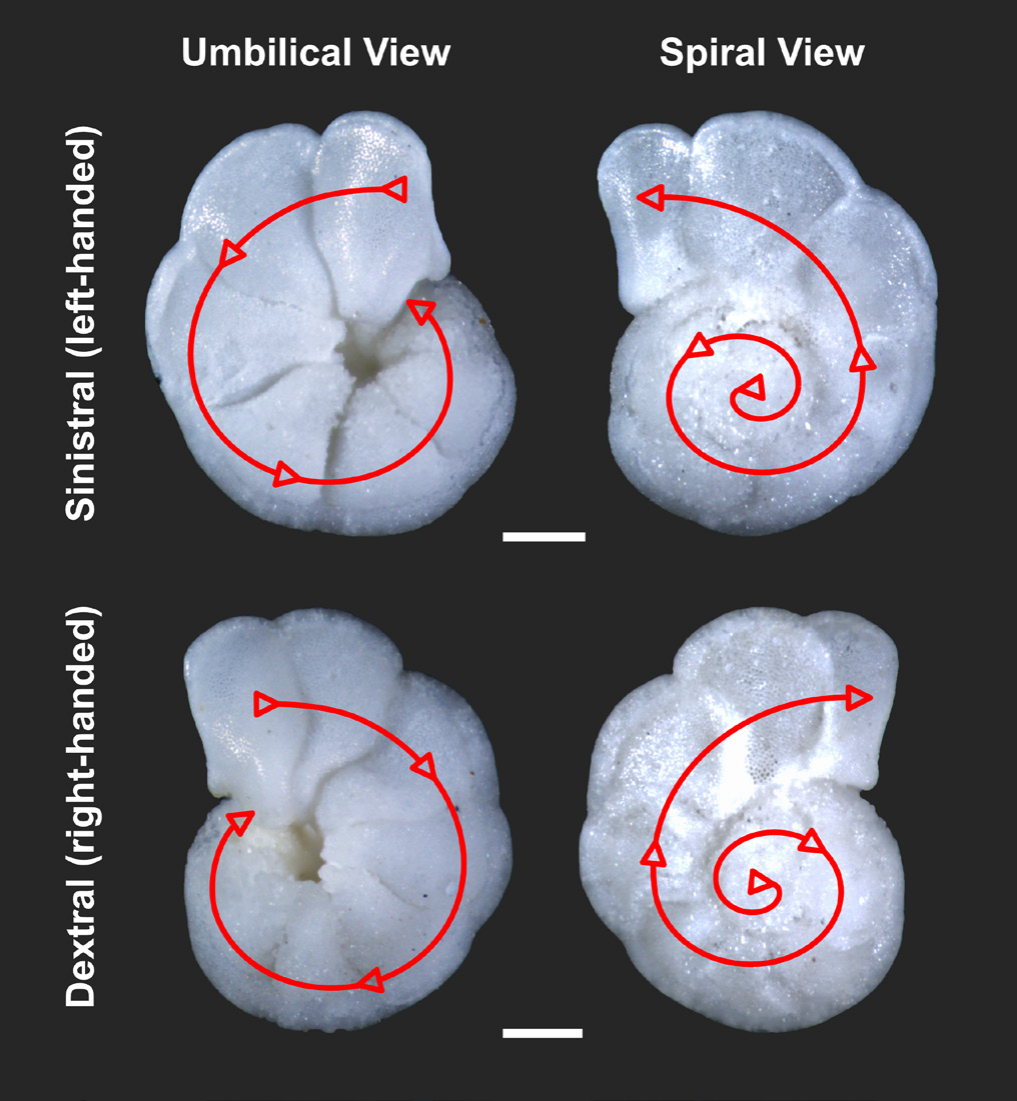
Schematic illustration of sinistral (counterclockwise) and dextral (clockwise) coiling arrangements in the species *Globorotalia limbata* in both an umbilical and spiral view. (Scale bar, 200 µm.)

In the 1950s, scientists studying ocean sediment cores in the North Atlantic noticed the species *Neogloboquadrina pachyderma* appeared to flip its dominant coiling direction back and forth. Populations were mostly counterclockwise (sinistral) when cold and clockwise (dextral) when the climate was warm (1). Initially, temperature was thought to be a determining effect, but later research revealed two distinct populations on either side of the polar front, each with its own coiling preference. As the climate cooled and ice advanced, so the water masses shifted south, bringing the sinistral population to places previously occupied by the dextrals.

Thanks to the International Ocean Discovery Program and its predecessor programs, we now have sediment cores from around the world and researchers can revel in a fossil record of unrivaled completeness stretching back tens of millions of years. The unfolding evolutionary narrative is drawing renewed attention, with coiling once again at the forefront, albeit with a different twist. The case studies discussed below differ from the *N. pachyderma* example in that the coiling changes were global. Temperature does not account for the pattern, because these coiling flips show up everywhere, from the tropics to the high latitudes, and during both warm periods and ice ages. So, what is the mechanism behind these flipping plankton?

## Results and Discussion

During a period of peak warmth known as the Early Eocene Climate Optimum (53 to 49 Mya), the long extinct genus *Morozovella* shifted from predominantly dextral to sinistral coiling soon after warming began (2). Sinistral shells show lower carbon isotope values, suggesting reduced reliance on photosymbiotic algae (2). It is likely that the sinistral shells are different genetic variants with distinct ecologies providing some kind of fitness advantage over the dextral forms. These differences may have enabled sinistral forms to tolerate the new warm environment.

Similar patterns occur in two other groups investigated in the Miocene epoch (~23 to 5.3 Mya), in nine sites, across different ocean basins and climate belts (3). A synchronous change from mixed to sinistral coiling in *Paragloborotalia siakensis* was found at 15.37 Mya, while the *Globorotalia scitula* lineage flipped twice: mixed to sinistral at 15.14 Ma, then sinistral to dextral at 10.02 Ma (3). It seems truly puzzling that a species could exist for millions of years coiling one way, and then suddenly reverse, for no apparent reason.

**Fig. 2.**
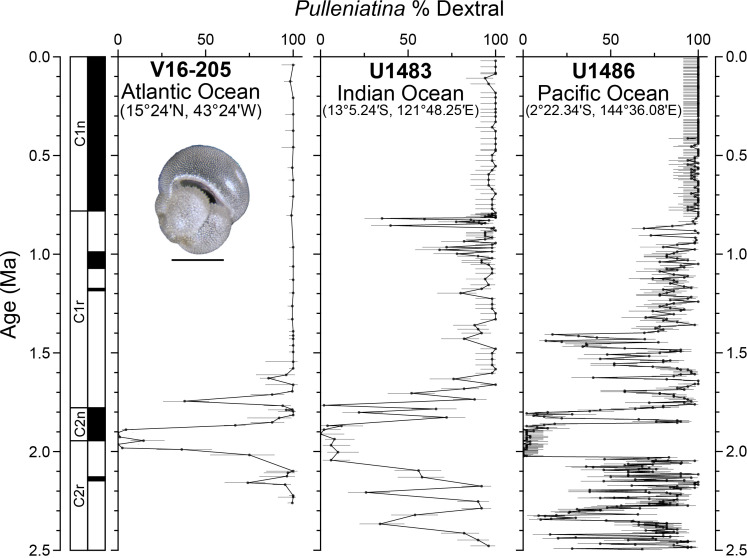
Summary of coiling variability in the planktonic foraminifera genus *Pulleniatina* over the past 2.5 Mya from several global oceanic sites. Error bars denote the 95% CI. Site V16-205 from ref. 6, sites U1483 and U1486 from ref. 5. Original data have been recalibrated to a common time scale (7) to allow for intersite comparison. Light microscope image of *Pulleniatina obliquiloculata* from ref. 8. (Scale bar, 200 µm.)

Another example is *Pulleniatina obliquiloculata*, which evolved 4.2 Mya (4) and still thrives in tropical oceans worldwide. For the past 860,000 y, it has been almost entirely dextral (Fig. 2), but before that there was a series of rapid shifts, sometimes resulting in sinistral or dextral dominance and other periods of mixed coiling (4, 5). These major shifts occurred worldwide and within a few thousand years. Geochemical data (oxygen and carbon isotopes) of hundreds of individual shells showed in some instances a divergence in average values between dextral and sinistral specimens, despite the size and shape being indistinguishable. This may indicate subtle habitat differences between the dextrals and sinistrals, or peak abundances at distinct times of year.

Important clues come from the genetics of modern populations, where cryptic diversity is common and the coiling direction seems to have a heritable genetic basis (9, 10). Although single celled, planktonic foraminifera reproduce sexually by releasing gametes, some are capable of asexual reproduction as well. It has generally been assumed that like most sexual species they evolve as one large group by mutation, recombination, and natural selection, with favorable genes spreading through the population and becoming fixed in time. But cryptic forms are sufficiently distinct that they have probably been established for a substantial amount of time.

Water masses function as dispersal barriers in the open ocean, partitioning habitats and limiting gene flow. In living *P. obliquiloculata*, for instance, several genetically distinct but morphologically identical variants exist, all exclusively dextral, whereas *Globorotalia truncatulinoides* includes a genotype with frequent sinistral forms. The cryptic genotypes show subtle but consistent habitat differences: One form of *P. obliquiloculata* is mostly found in the warmest part of the west Pacific, while the others occur more widely (9). The pattern is even more marked in *G. truncatulinoides*, where the habitats are geographically partitioned, with various forms dominating different water masses (10).

These cryptic populations could explain those puzzling coiling shifts. What if species like *P. obliquiloculata* are continually producing slightly different variant forms, each largely isolated in a reproductive sense but prospering or dwindling depending on circumstances? In time, such forms might diverge slightly and coexist, but every now and again one could achieve a global selective advantage and replace the rest. Most of the time that sort of replacement would be invisible to the fossil record, but occasionally it might involve forms with different coiling preferences, possibly having established themselves in small founder populations. Then, and only then, would we see a sudden flip in the sediment record. The term for this kind of evolutionary replacement is a “population sweep,” and it is most familiar to biologists as the way asexual organisms like bacteria evolve when confronted with new selective pressures. Viruses, too, have a similar pattern, as we all became aware of during the Covid-19 pandemic when variants evolved, spread widely, and got replaced. And if the variant forms were not so different from each other, the result may simply be a change in a few genes. Returning to *N. pachyderma*, genetic studies proved that the sinistral and dextral variants differ so much that the dextral form, which also has subtle morphological differences, is now regarded as a separate species *N. incompta* (11). It seems that in some cases, the variants might diverge long enough, adapting to different water masses and conditions.

So, what, in all this, is a species? Of course, that depends partly on which definition one adopts, but there is also an interesting question involved, not just a semantic one. At least in the case of the planktonic foraminifera, it seems reproductive isolation alone is not sufficient to produce lasting divergence. Instead, the concept of *ecological speciation* is supported, wherein the separation of habitat niches appears crucial for generating biodiversity. Finding a new way of life is evidently more difficult than establishing barriers to reproduction, at least for the forams.

If this thinking is right, coiling direction itself may offer no inherent advantage: Instead, it serves as an accidental marker for hidden genetic and ecological differences. But that does not explain *why* a particular population should favor clockwise or counterclockwise coiling. The shell is constrained by geometry to go one way or the other, even though single-celled organisms themselves have no obvious handedness. Why certain genes favor left or right coiling remains a mystery. It is possible that genetics, reproductive mode, and environmental conditions all have an influence as the organism starts to build its microscopic spiral. Thus, now we await advances at the molecular level to resolve the enigma of these flipping plankton.

## Materials and Methods

We integrated and synthesized case studies from published sources. *Globorotalia limbata* specimens (Fig. 1) are from Lo-En Guyot, western Pacific Ocean, Ocean Drilling Program Hole 872C, 4H-1, 59-61 cm (28.59 m below sea floor), Upper Miocene Subzone N17b (=Zone M13b-M14). Light microscope imaging was conducted using an Olympus SZX16 stereo microscope, equipped with a DP73 multifocal camera in the Department of Earth Sciences, University College London. Image stacking was performed using the Stream Motion (Olympus) software.

## Data Availability

Previously published data were used for this work (2–6).
